# Effects of 5-Fluorouracil on Morphology, Cell Cycle, Proliferation, Apoptosis, Autophagy and ROS Production in Endothelial Cells and Cardiomyocytes

**DOI:** 10.1371/journal.pone.0115686

**Published:** 2015-02-11

**Authors:** Chiara Focaccetti, Antonino Bruno, Elena Magnani, Desirée Bartolini, Elisa Principi, Katiuscia Dallaglio, Eraldo O. Bucci, Giovanna Finzi, Fausto Sessa, Douglas M. Noonan, Adriana Albini

**Affiliations:** 1 Science and Technology Center, IRCCS MultiMedica, Milan, Italy; 2 Department of Research and Statistics, IRCCS Arcispedale Santa Maria Nuova, Reggio Emilia, Italy; 3 Oncology Unit, IRCCS MultiMedica, Castellanza, VA, Italy; 4 Department of Pathology, Ospedale del Circolo, Varese, Italy; 5 Department of Surgical and Morphological Sciences, University of Insubria, Varese, Italy; 6 Department of Biotechnology and Life Sciences, University of Insubria, Varese, Italy; National Cheng Kung University, TAIWAN

## Abstract

Antimetabolites are a class of effective anticancer drugs interfering in essential biochemical processes. 5-Fluorouracil (5-FU) and its prodrug Capecitabine are widely used in the treatment of several solid tumors (gastro-intestinal, gynecological, head and neck, breast carcinomas). Therapy with fluoropyrimidines is associated with a wide range of adverse effects, including diarrhea, dehydration, abdominal pain, nausea, stomatitis, and hand-foot syndrome. Among the 5-FU side effects, increasing attention is given to cardiovascular toxicities induced at different levels and intensities. Since the mechanisms related to 5-FU-induced cardiotoxicity are still unclear, we examined the effects of 5-FU on primary cell cultures of human cardiomyocytes and endothelial cells, which represent two key components of the cardiovascular system. We analyzed at the cellular and molecular level 5-FU effects on cell proliferation, cell cycle, survival and induction of apoptosis, in an experimental cardioncology approach. We observed autophagic features at the ultrastructural and molecular levels, in particular in 5-FU exposed cardiomyocytes. Reactive oxygen species (ROS) elevation characterized the endothelial response. These responses were prevented by a ROS scavenger. We found induction of a senescent phenotype on both cell types treated with 5-FU. In vivo, in a xenograft model of colon cancer, we showed that 5-FU treatment induced ultrastructural changes in the endothelium of various organs. Taken together, our data suggest that 5-FU can affect, both at the cellular and molecular levels, two key cell types of the cardiovascular system, potentially explaining some manifestations of 5-FU-induced cardiovascular toxicity.

## Introduction

The antimetabolite 5-Fluorouracil (5-FU), an analogue of uracil, and its pro-drugs are widely used antineoplastic agents for the treatment of gastrointestinal cancers, breast, gynecological as well as head and neck tumors [1]. 5-FU availability for intracellular anabolism mainly depends on tissue drug catabolism. After administration, 5-FU follows different metabolic destinations: more than 80% of the dose is inactivated by biotransformation primarily in the liver, approximately 15–20% is eliminated in the urine and only a small fraction remains available to exert its anti-tumor action [2].

Capecitabine (N4-pentyloxycarbonyl-5'-deoxy-5-fluorocytidine), an orally administered fluoropyrimidine carbamate 5-FU prodrug, is converted into 5-FU through sequential steps (S1 Fig.) with preferential activation in tumors because of tissue distribution of key metabolic enzymes, in particular Thymidine phosphorylase (TP) [3]. 5-FU acts during the S phase of the cell cycle inhibiting DNA synthesis by restricting availability of thymidylate (S1 Fig.). TP is also a key enzyme for production of the 5-FU active metabolite [4]. 5-Fluorouracil inhibits thymidylate synthetase through its metabolite 5-fluorodeoxyuridine monophosphate (FdUMP). FdUMP forms a covalent ternary complex with thymidylate synthetase and 5,10-methylene tetrahydrofolate. Association with folinic acid increases the stability of the complex. 5-FU can also inhibit RNA synthesis, processing and function [4,5] (S1 Fig.). TP is expressed at low levels in many tissues throughout the body [6], and at high concentrations in most tumor tissues, leading to the accumulation of 5-FU in tumors [4,7]. Pharmacokinetic studies performed on intravenous bolus 5-FU single dose show that maximum plasma concentrations of 5-FU can reach a millimolar range with a subsequent rapid decline [8–10]. The non-linearity of 5-FU kinetics probably reflects the saturation level of metabolic processes or transport at the highest concentrations of the drug and represents the main reason justifying the difficulty in predicting the plasma levels or toxicity at high doses [10].

The preferential tumor-accumulation of fluorouracil-based drugs within tumor tissues favors tolerability, however side effects can occur. Leukopenia, diarrhea, stomatitis, and nausea manifest frequently in patients treated with 5-FU, while hand-foot syndrome is a typical side effect of Capecitabine [11]. Cardiac toxicity of fluoropyrimidines, which can be severe and life threatening, is the second most common cause of chemotherapy-induced cardiotoxicity [12]. Clinical cardiac toxicities associated with 5-FU covers a wide range of manifestations: coronary vasospasms and subsequent calcium antagonist non-responding angina, myocardial infarction, ischemia, dysrhythmia, cardiomyopathy, tako-tsubo cardiomyopathy, sinoatrial and atrioventricular nodal dysfunction, QT prolongation with torsades de pointes ventricular tachycardia, cardiac arrest and sudden death have been reported in the literature [12–21]. Heart failure is reported in 3.5% of patients, often during the first cycle of chemotherapy [22] and specific side effects occur when 5-FU is administered either as a single agent or in combination with intermediate-dose folinic acid [23]. Recent meta-analyses for fluoropyrimidine cardiotoxicity not only indicates incidence ranging from 0–20% for 5-FU and 3–35% for Capecitabine [20,21], but also highlighted an increased risk of toxicity during administration of higher doses of 5-FU as well as of concomitant cisplatin treatment, or of continuous infusion as compared to bolus administration. Subclinical cardiac toxicity may be much higher. In a prospective study, 29% of patients receiving 5-FU had evidence of cardiac toxicity (N-terminal probrain natriuretic peptide elevation, a marker of left ventricular dysfunction) [22]. Difference in incidence of cardiotoxicity between studies could be a reflection of different risk profiles [20]. In a multivariate analysis patients with preexisting cardiovascular disease have a relative risk of 8 for cardiotoxicity compared to patients without cardiac disease [22]. A high rate of recurrence of cardiac toxicity after rechallenge is reported, varying from 20% to 100% [24,25]. A literature review indicated an overall death rate of 0.32% for first administration, whereas re-exposure of those subjects who previously had cardiac toxicity led to 13% death rate, 40-fold higher [26].

Some 5-FU metabolites are also associated with toxicity, in particular alpha-fluoro-beta-alanine has been associated with neuro- and cardiotoxicity [27]. Thymidine phosphorylase (TP) is a key enzyme involved in conversion of Capecitabine to 5-FU and 5-FU to its active metabolites. TP was also found to be an angiogenic factor (known as platelet-derived endothelial cell growth factor) [28,29]. TP expression is up-regulated in atherosclerotic plaques [30] and during myocardial infarction [31], potentially contributing to the higher prevalence of cardiotoxicity in patients with previous cardiovascular disease or 5-FU induced damage.

Follow up studies have shown that a key point to take into consideration is that the time of cardiotoxicity clinical manifestation is widely variable, eventually being immediate or at late onset, years or decades after treatment [16–18,32,33] and more importantly this risk is individual [16–18,32]. The incidence and severity of cardiac events depend on the type of drugs used, the dose and the schedule employed as well as on patient age, presence of coexisting cardiac diseases or previous mediastinal irradiation [16–18,32]. Acute cardiac toxicity often can be prevented by interrupting treatment when the patient is close to the maximum cumulative dose, while long-term effects are difficult to predict since they become apparent long after chemotherapy, often as a result of a stressful event, surgery, pregnancy or changes in lifestyle [34,35].

In terms of cardiotoxicity, the most well studied anti-tumor agents are anthracyclines, trastuzumab, and their combination [17,36,37]. Less is known about the mechanisms of fluoropyrimidine induced cardiotoxicity. Among the numerous possible effects [12,21] current hypotheses involve: 1) An imbalance between anabolic and catabolic processes during biotransformation, leading to the considerable inter-individual variability [1]. 2) A direct 5-FU mediated damage to the vascular endothelium, followed by thrombosis, characterized by the release of vasoactive substances and vasospams or the alteration of the antioxidant defense capacities in myocardial tissues, due to the exhausted activity of the cardiac enzymes superoxide dismutase and glutathione peroxidase [13,14]. In few cases coronary artery vasospasm and vasoconstriction has been visualized during coronary angiography immediately after 5-FU injection [17,36,37], while cardiotoxicity may occur at the end of infusion or hours to days later [38].

In order to develop effective cardiotoxicity prevention strategies, a deeper and conclusive knowledge of the causes of these toxicities is needed. The effects of fluoropyrimidines have been investigated on several cell types (tumor and normal cells) including smooth muscle cells [39], and on numerous animal models, but the effects of 5-FU on human endothelial cells and cardiomyocytes has, to our knowledge, not been previously investigated. Given the broad range of cardiac toxicities associated with 5-FU [12,21], these studies are needed to help understand the effects on the cardiovascular system [38]. To shed light on some of the mechanisms of 5-FU cardiotoxocity, here we examined the effects of 5-FU on endothelial cells and cardiomyocytes *in vitro* and in a colorectal cancer xenograft model, analyzing both at cellular and molecular levels the mechanisms involved in cardio-vascular toxicity associated with 5-FU treatment.

## Materials and Methods

### Cells and reagents

Human umbilical vein endothelial cells (HUVECs) (Promocell, Heidelberg, Germany) were grown on 1% gelatin-coated tissue culture plates in M199 growth medium (Sigma-Aldrich, St Louis, MO, USA), supplemented with heat inactivated 10% FBS (Sigma-Aldrich), 2 mM glutamine (Gibco-Life Technologies, Thermo Fisher Scientific, Waltham, MA, USA), 100 μg/ml heparin sodium salt (Sigma-Aldrich), 10 μg/ml hydrocortisone (Sigma-Aldrich), 10 ng/ml endothelial growth factor (EGF), 10 ng/ml acid and basic fibroblast growth factor (aFGF, bFGF) (Peprotech, Rocky Hill, NJ, USA). Human cardiac myocytes (HCMs, Promocell) were grown in Supplemented Myocyte Growth Medium (Promocell). Primary cell cultures were maintained at 37°C and 5% CO_2_ and were used in all *in vitro* experiments between passages 3 and 8.

Human colorectal cancer (CRC) cell lines HCT-116 and HT29 (both from ATCC) were cultured on McCoy medium (Gibco-Life Technologies), murine CT26 CRC cells (ATCC) were cultured in RMPI1640 medium (Gibco-Life Technologies), all supplemented with heat inactivated 10% FBS (Sigma-Aldrich), 2 mM glutamine (Gibco-Life Technologies) and 1% penicillin/streptomycin (Invitrogen, Life Technologies).

5-Fluorouracil (5-FU), 1-(4,5-dimethylthiazol-2-yl)-3,5-diphenylformazan (MTT), the Toxicology Assay Kit (TOX7), Propidium Iodide (PI), the Senescence Cells Histochemical Staining Kit, 2',7'-dichlorodihydrofluorescein-diacetate (DCFH-DA), N-acetyl-L-cysteine (NAC), Acridine Orange (AO) and Vincristine were purchased from Sigma-Aldrich. The FITC BrdU flow kit, Matrigel, Annexin V and 7AAD were purchased from BD Biosciences (San Jose, CA, USA).

### Cell proliferation assay

HUVECs were seeded on 1% gelatin coated tissue culture plates at 2x10^3^ cell/well in 96-well plate. HCMs were plated at the same density in their specific growth medium. HCT-116 and CT26 were seeded at 1x10^3^ cells/well, HT29 at 3x10^3^ cells/well, in 96-well plates. After a 24 hour incubation in their respective media to allow for cell attachment, the medium was changed and the cells were treated with serial 10-fold dilutions (10 nM to 1 mM) of 5-FU dissolved in DMSO. As negative controls, growth medium with DMSO alone were used, 0.1% saponin was the positive control. All conditions were incubated for up to 96 hours and 6–8 replicates were done for each time-point. The MTT reagent was added to a final concentration of 0.5 mg/ml to each well. After 5 hours incubation at 37°C, medium was removed, formazan crystals were dissolved with 100 μl of DMSO and absorbance read at 570 nm in a micro-plate reader. Non-viable cells are unable to reduce the MTT dye, giving an indirect measure of 5-FU effects on cell number. The concentration of drug that reduced cell proliferation by 50% (EC_50_) was calculated by non-linear regression fit using GraphPad Prism.

### Detection of apoptosis by flow-cytometry

The proportion of apoptotic cells was detected by the Annexin V / 7AAD staining. HUVE or HCM cells were plated at a density of 5x10^4^ cells/well on 6-well plates and grown overnight. The subsequent day the cells were treated with increasing 5-FU concentrations. Controls received DMSO (negative) or Vincristine (positive). Each treatment was in triplicate and three different experiments were performed. After 96 hours, cells were harvested, counted, transferred into flow tubes, pelletted, resuspended in 100 μl of fresh 1X Annexin binding buffer (0.01 M HEPES pH 7.4; 0.14 M NaCl; 2.5 mM CaCl_2_) plus Annexin V fluorescein isothiocyanate (FITC) and 7AAD peridinin-chlorophyll protein (PerCP). After staining, samples were analyzed by flow cytometry within 1 hour using a FACSCanto (BD). Annexin V^+^/7AAD^±^ were considered as apoptotic cells. The proportion of apoptotic cells was expressed as a percentage of the total cell number acquired, excluding debris, and analyzed using the BD FACSDiva and FlowJo software.

### Cell cycle analysis

Assessment of cell proliferation was evaluated both with classic PI staining and through direct measurement of DNA synthesis using incorporation of the nucleoside analog bromodeoxyuridine (BrdU). For PI staining, cells were plated on 6-well plates, grown overnight, treated in triplicate with the same concentrations and controls as above. Treatments were continued up to 96 hours and each well was collected singularly into flow tubes. Cells were fixed and permeabilized with 70% cold ethanol for at least 1 hour at -20°C, then washed twice with cold Phosphate Buffer Solution 1X and stained for 40 minutes at 37°C with PI solution (50 μg/ml PI in H_2_O, 0.1% Triton-X100, 0.1% trisodium citrate dehydrate, 6.25 μg/ml RNase A). Cells were analyzed by flow cytometry within 3 hours using a FACSCanto machine (BD) and analyzed with BD FACSDiva Software 6.0.

For BrdU staining, asynchronized log-phase growing cells (40% confluent) were treated 96 hours with 5-FU (100 nM—1 mM) or with DMSO in complete medium. As a positive control 50 nM Vincristine, known inhibitor of cell cycle, was added as a positive control 48 hours before the end of treatment. BrdU (10 μM) was added 12 hours before the end of the experiment, thus only cells still capable of replication following 5-FU treatment are able to incorporate BrdU. BrdU incorporation was measured using an anti-BrdU FITC-conjugated antibody and detected by flow cytometry along with 7ADD staining for total DNA.

### LDH assay

Lactate dehydrogenase (LDH) is an intracellular enzyme that is released in the tissue culture supernatant when cell membranes are damaged, and it is also a marker for cardiac myocyte injury [40]. HUVECs and HCMs were plated on 96 well plates, as previously seen and exposed for 96 hours to varying concentrations of 5-FU, DMSO alone or 0.1% saponin (negative and positive controls, respectively). Lactate dehydrogenase release was detected using the TOX7 (Sigma-Aldrich) colorimetric assay according to the manufacture’s instructions.

### Ultrastructural analysis

For ultrastructural analysis, HUVE and HCM cells were treated with 1 mM 5-FU for 72 or 96 hours. Growth media alone and containing DMSO were used as controls. Cells were fixed in a mixture of 2% paraformaldehyde and 2% glutaraldehyde, post-fixed in 1% osmium tetroxide, and embedded in Epon-Araldite (Sigma-Aldrich). After counterstaining with uranyl acetate and lead citrate, thin sections were examined with a Morgagni electron microscope (Philips, Eindhowen, NL) at 7100X magnification.

### Reactive Oxygen Species (ROS) detection

ROS detection protocol was adjusted from that described previously [41]. HUVECs and HCMs were plated on 6-well plates, at 5x10^4^ cells/well. The following day, cells were treated with serial dilutions of 5-FU (10 nM—1 mM). DMSO and N-(4-hydroxyphenyl)retinamide (4HPR, Sigma-Aldrich) were used as controls. After 96 hours cells were harvested, centrifuged and washed in PBS and loaded with 50 μM 2',7'-dichlorofluorescein-diacetate (DCFH-DA) for 10 minutes at 37°C. Fluorescence was detected with a FACSCanto cytometer (BD) on the FL1 channel and analyzed with FACSDiva 6.0 software. In some experiments the ROS scavenger NAC (10 mM) was added concomitant with 5-FU treatments.

### Detection of Acidic Vesicular Organelles (AVOs)

Cells undergoing autophagy develop double-membrane, acidic vesicular organelles (autophagosomes, AVOs), which can be detected by specific dyes, in particular acridine-orange (AO). AVOs were quantified by flow cytometry after staining of cells with AO. Cells were plated and treated as above in 6-well plates for 96 hours, then harvested, collected in flow tubes and washed in PBS. The cell suspension was stained with AO (5 μg/ml) for 15 min at room temperature, then washed twice in PBS, resuspended in PBS and analyzed by flow cytometry. Fluorescence was read on the FL2 channel (PE) on a FACSCanto cytometer (BD) and analyzed with FACSDiva 6.0 software. The AO bright red fluorescence intensity is proportional to the degree of acidity and correlate with AVOs formation. In some experiments the ROS scavenger NAC (10 mM) was added concomitant with 5-FU treatments.

### Immunocytochemistry for LC3 localization

Endothelial cells and cardiomyocytes were separately plated at 5 × 10^3^ onto 8-well chamber slides (Nunc, Thermo Fisher Scientific) and treated with 5-FU as above, washed with PBS and fixed with 4% paraformaldehyde at 37°C for 30 min. Cells incubated with DMSO and 10 μM Cloroquine served as negative and positive controls. Cells were permeabilized with 0.5% Triton X-100 for 8 hours at 4°C and blocked with 0.2% bovine serum albumin (BSA) for 1 hour at room temperature. The cells were labeled with polyclonal rabbit anti-human LC3 (Cell Signaling Technology, Beverly, MA, USA) overnight at 4°C, and with Alexa Fluor 555 goat anti-rabbit secondary antibody (Invitrogen, Thermo Fisher Scientific) for 2 hours at room temperature in the dark. Images were acquired at 63X magnification with immersion oil under a Zeiss (Zeiss, Jena, Germany) fluorescent microscope.

### Western Blotting Analysis

Western blotting was performed with a SDS-PAGE Electrophoresis System. Cells were treated with 5-FU for 96 hours, then collected by plate scraping on ice. Total lysates were prepared using Cell Lysis Buffer (Cell Signaling Technology) and protein concentrations evaluated by the DC Protein Assay (Bio-Rad, Hercules, CA, USA). Equal amounts of proteins for each sample were resuspended in reduced sample buffer, resolved on a 12% SDS-PAGE and blotted onto polyvinylidene fluoride (PVDF) membranes (Amersham Biosciences, Otelfingen, Switzerland). Following non-specific blockage with 5% non-fat milk powder (w/v) in Tris-buffered saline (10 mM Tris–HCl, pH 7.5, 100 mM NaCl, 0.1% Tween-20) for 1 hour at room temperature, membranes were probed with polyclonal primary antibody rabbit anti-human LC3A/B antigen (Cell Signaling). The antibody was diluted in 5% bovine serum albumin-0.1% Tween-Tris-buffered saline according to the manufacturer’s instructions. Horseradish peroxidase-conjugated secondary antibodies were then added and the resulting signal detected through autoradiography using chemiluminescence (ECL, Amersham Biosciences). Rabbit anti-human anti-tubulin antibody (Cell Signaling), used according to the manufacturer instructions, served as a reference for normalization.

### Analysis of senescence induction

HUVECs or HCMs were seeded at 5x10^3^ cells/chamber in an 8-well chamber slide and treated with 5-FU up to 72 hours. Cells were then processed using a Senescence Cells Histochemical Staining kit (Sigma-Aldrich) as indicated. Briefly: media was removed and cells were washed in PBS, fixed and stained with a β-galactosidase reagent. Chamber slides were incubated over night at 37°C without CO_2_ and then observed in bright field and fluorescence after mounting with DAPI containing Vectashield Mounting Medium (Vector Laboratories, Burlingame, CA, USA). The ratio between the β-galactosidase positive blue-stained cells (bright field) and total amount of DAPI stained nuclei (fluorescence) was calculated per each observed field and the results reported as mean of percentages of senescent cells per treatment.

### Evaluation of 5-FU cardiovascular toxicity *in vivo*

CT26 murine colon cancer cells (1x10^6^) were suspended in 100 μl of matrigel (BD) and subcutaneously injected into the flanks of 6–8 week old BALB/c mice (Charles River Laboratories, Calco-Lecco, Italy). The animals were treated by intraperitoneal injection with either 10 mg/Kg 5-FU every 48 hours or 1 mg/Kg 5-FU every day, or vehicle alone, starting when tumors were palpable. Tumor dimensions were measured by a caliper in two dimensions, the tumor volume (mm^3^) was calculated using the formula width^2^ × length/2. After 20 days, mice were sacrificed, organs and tumors were collected, fixed overnight in 4% paraformalaldeyde and stored in 70% ethanol. For transmission electron microscopy, tissues were then post-fixed in 1% osmium tetroxide, and embedded in Epon-Araldite (Sigma-Aldrich), and processed for electron microscopy as above. For light microscopy, tissues were embedded in paraffin and sections stained with hematoxylin and eosin using standard protocols. All animals were housed in a conventional animal facility with 12 hours light/dark cycles and fed *ad libitum*. The use of animals was in accordance with the Italian and European Community guidelines (D.L. 2711/92 No.116; 86/ 609/EEC Directive) and approved by the institutional ethics committee. Groups of 4–8 mice were used for each treatment.

### Statistical analysis

Statistical analyses were performed using Prism software (GraphPad), two-tailed t-tests for comparison of two data sets and one-way or two-way ANOVA for multiple data sets were applied, P<0.05 was considered statistically significant. Means ± SEM of experiments performed in triplicate or quadruplicate are shown.

## Results

### Effects of 5-FU on endothelial cells and cardiomyocyte cell growth and survival

**Figure 1:**
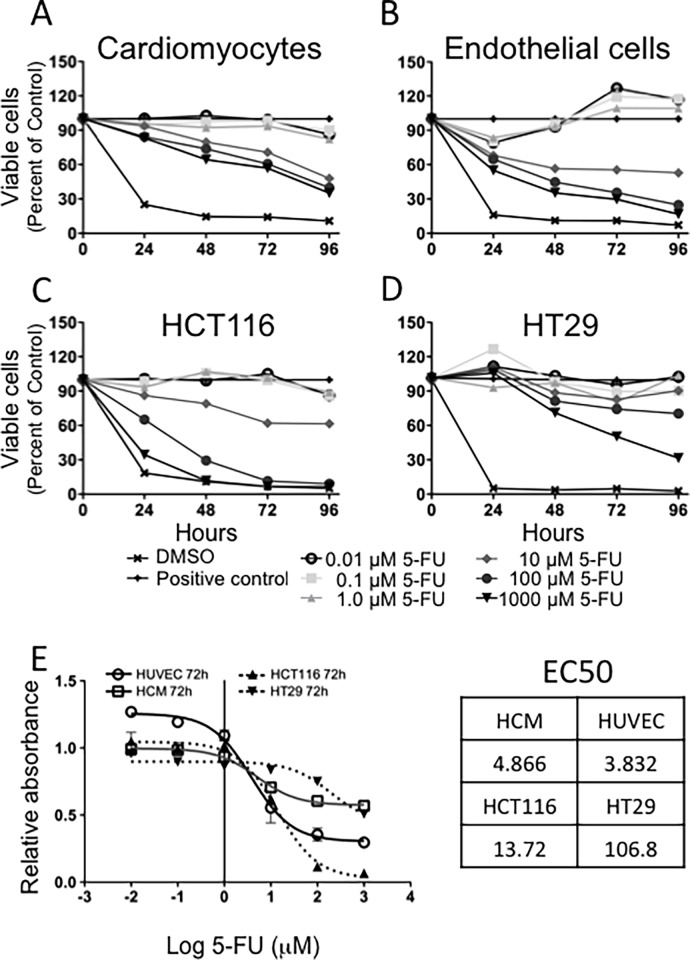
Cytostatic/cytotoxic effects of 5-FU on cardiomyocytes and endothelial cells. The cytostatic effect of 5-FU on HCMs (A) was not marked but statistically significant starting at 48 hours and from 10 μM 5-FU. The inhibitory effect of 5-FU was significant in endothelial cells (B) already after 24 hours at the intermediate concentration of 10 μM. The inhibition of cell viability was maintained up to 96 hours. Drug efficacy was also tested on the HT29 and HCT116 colorectal cancer cell lines (C, D). Mean ± S.E.M. of four different experiments for each cell type is shown. (E) MTT data at 72 hours were used to calculate the EC_50_ values using the non-linear regression function of GraphPad Prism. 5-FU concentrations are reported in μM on a Log_(10)_ scale, DMSO: 0.2% DMSO (vehicle) negative control, 0.1% saponin, positive control.

We initially evaluated the effects of 5-FU on HCM (Fig. 1A) and HUVE (Fig. 1B) cell proliferation using a standard MTT assay. Inhibition of endothelial cell growth became statistically significant at 10 μM or higher concentrations of 5-FU already after 24 h, and was maintained throughout the entire time-course. Doses lower than 10 μM did not show cytostatic effects. A similar response to 5-FU treatment was found for HCM: 10 μM or higher concentrations exerted cytostatic effects, lower concentrations of 5-FU did not influence cell proliferation. We compared the effects on vascular cells to the therapeutic outcomes exerted on colon cancer cells. Comparison of 5-FU efficacy on colon cancer cell lines (HCT116, HT29; Fig. 1C, D) confirmed a response, with half maximal effective concentration (EC_50_) of 13.72 μM for HCT116 and 106.8 μM for HT29 cells (Fig. 1E). Side effects on vascular cells occurred at EC_50_ of 3.832 μM for HUVECs and 4.866 μM for HCMs. Cellular replication rates varied widely between cardiomyocytes, endothelial and tumor cells (S2 Fig.).

**Figure 2:**
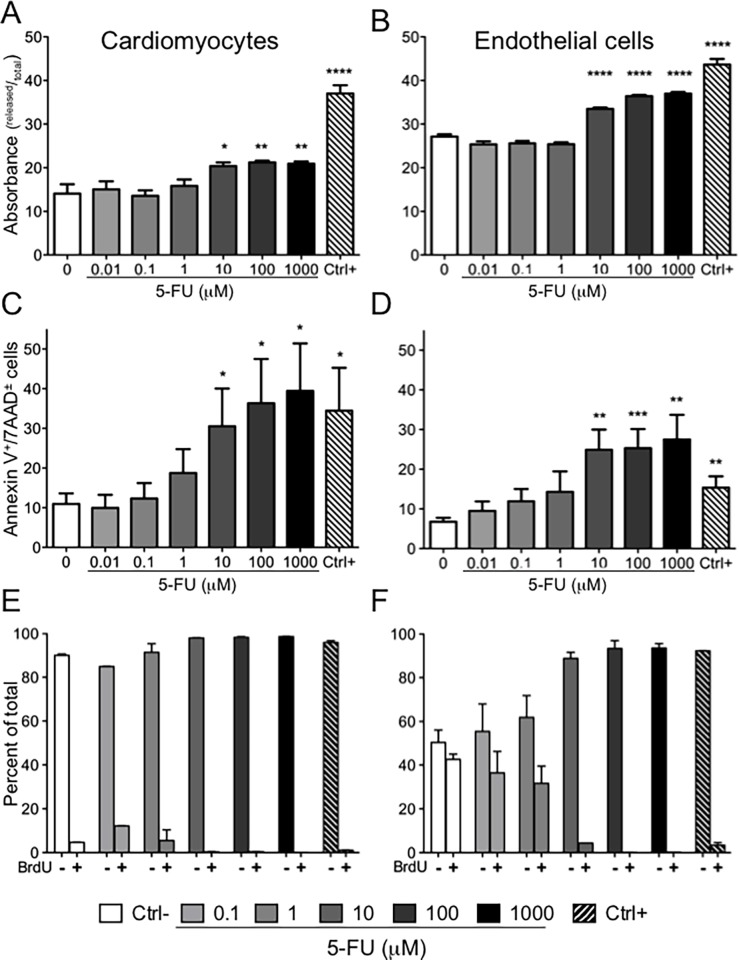
Effects of 5-FU on HCMs and HUVECs integrity and cycling ability. Membrane damage was calculated as the ratio between released LDH and total LDH. The effect of 5-FU on LDH release was statistically significant at 5-FU concentrations higher that 10 μM, less marked on cardiomyocytes (*P<0.05; **P<0.001) (A) with respect to HUVECs (****P<0.0001) (B). Mean ± S.E.M. of three different experiments for each cell type is shown. After 96 hours of 5-FU treatment cells were collected and stained with Annexin-V/7AAD to determine induction of apoptosis. Cumulative graphs of three independent experiments showing Annexin V^+^/7AAD^±^ cells are shown. Higher concentrations induced statistically significant amount of apoptotic cells as compared to negative control both in HCM (* P<0.05) (C) and HUVEC (**P<0.01; ***P<0.005) (D). Impairment of proliferative capacity was assessed using BrdU incorporation after 84 hours of treatment with 5-FU. BrdU-unlabeled cells (BrdU^-^) represent cells in G1 and G2/M phases, BrdU-labeled cells (BrdU^+^) represent replicating cells in S phase. Staining of HCMs (E) revealed a certain degree of replicating capability in the negative control and at the lower concentrations of 5-FU, with complete absence of cells in the BrdU^+^ fraction in doses of 10 μM or more. Endothelial cells (F) display a dose dependent accumulation of BrdU^-^ cells in G0 in the presence of 5-FU. Mean ± S.E.M. of three different experiments for each cell type is shown. Negative controls (Ctrl-) consisted of cells treated with vehicle alone (0.2% DMSO), positive controls (Ctrl+) were cells treated with 0.1% saponin.

Initial cell cycle analysis using PI staining indicated a trend toward an increase in apoptotic cells and a decrease in replicating (S phase) endothelial cells with 5-FU treatments greater than 1 μM (S3A Fig.). We further investigated the 5-FU cytostatic/cytotoxic effects using the Lactic Dehydrogenase (LDH) release assay, which measures loss of membrane integrity through detection of LDH enzyme activity in the culture medium. 5-FU concentrations greater than 1 μM significantly compromised cell membrane integrity for both cardiomyocytes and endothelial cells (Fig. 2A, B). We examined in depth the ability of 5-FU to induce apoptosis using Annexin V and 7AAD staining (Fig. 2C, D). After a prolonged (96 hour) treatment with 5-FU, both cell types showed 5-FU induced cell death (Annexin V^+^/7AAD^±^) (S4 Fig.) with percentage of apoptosis induced in cardiomyocytes and endothelial cells statistically significant (P<0.05) for concentrations of 10 μM, 100 μM and 1 mM (Fig. 2C, D).

5-FU effects on the cell cycle were further examined using an assay where cellular DNA content was directly labeled with 7AAD while proliferative capacity following 84 hours of treatment with 5-FU was assessed by a 12 hours incorporation of BrdU (Fig. 2 E, F). Two gates were set on the BrdU/7AAD cytograms (S3B Fig.) according to BrdU content: BrdU-unlabeled (BrdU^-^) cells in the bottom gate represent cells in G1 and G2/M phases, while BrdU-labelled cells (BrdU^+^) in the upper gate represent replicating cells in S phase. Staining of HCMs revealed a certain degree of replicating capability in the negative control and at the lower concentrations of drug (100 nM and 1 μM, light grey bars, Fig. 2E). Treatment with 5-FU revealed high sensitivity with complete absence of cells in the BrdU^+^ fraction at doses of 1 μM or higher (Fig. 2E). Similarly, endothelial cells showed a dose dependent accumulation of BrdU^-^ cells in G1 and G2/M in the presence of 5-FU (Fig. 2F). Cells in the BrdU^+^ fraction decreased from 42.6% in the negative control to 36.5% at 100 nM, 31.7% at 1 μM and 4.4% at 10 μM and were undetectable at 100 μM and at 1000 μM 5-FU.

### 5-FU induces autophagy of endothelial cells and cardiomyocytes

**Figure 3:**
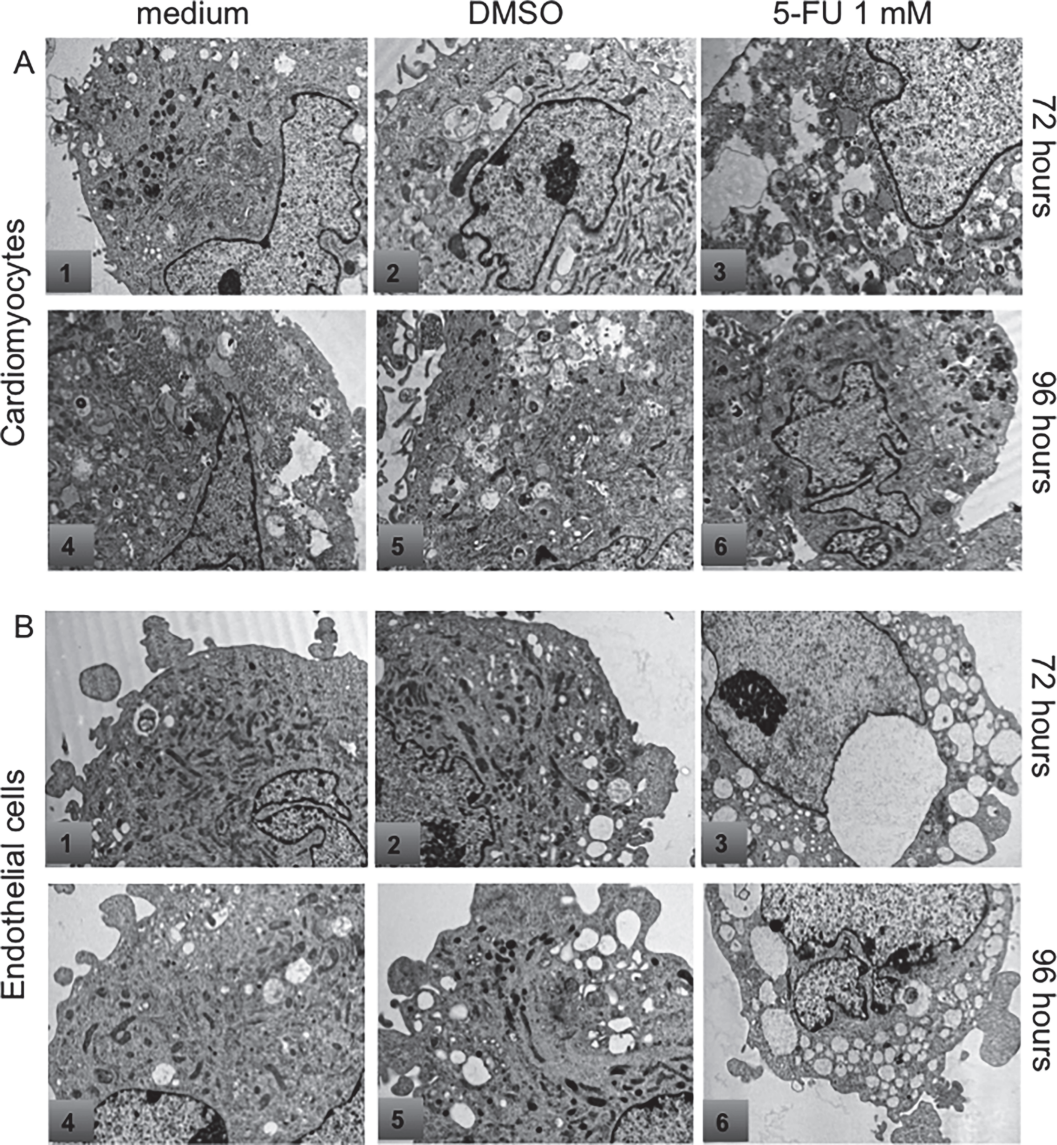
Ultrastructural analysis by electron microscopy. HCMs in culture without any treatment (A 1, 4) or treated with DMSO (A 2, 5) showed only very mild signs of distress. HCMs treated with 1 mM 5-FU for 72 and 96 hours (A 3, 6) showed signs of suffering including rupture of the mitochondrial christae, dilatation of reticulum cysternae and autophagic vacuoles. HUVEC in culture without any treatment (B 1, 4) or treated with DMSO (B 2, 5) did not show any signs of mitochondrial rupture. HUVEC treated with 1 mM 5-FU for both 72 and 96 hours (B 3, 6) showed several signs of injury (rupture of mitochondrial christae and dilatation of reticulum cysternae).

**Figure 4:**
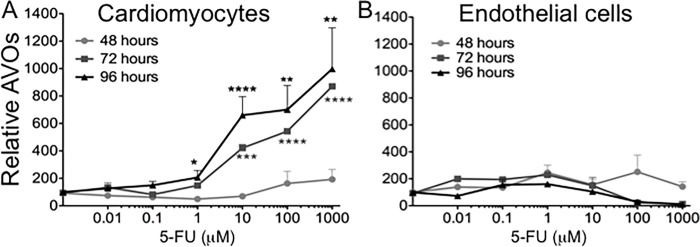
Acidic Vesicular Organelles (AVOs) formation in HCM after 5-FU treatment. AVOs formation is associated with the establishment of an autophagic process. Prolonged treatment with 5-FU significantly increased AVOs accumulation (*P<0.05; **P<0.01; ***P<0.005; ****P<0.0001) in HCM (A) as compared to control. Both 48 as well as 96 hours treatment of HUVEC (B) did not significantly influence the amount of acidic compartment staining over time. Mean ± S.E.M. of four different experiments per cell types are shown.

Transmission electron microscopy analysis of 5-FU treated endothelial cells and cardiomyocytes showed clear signs of autophagy (Fig. 3). Both HCM and endothelial cells treated with 1 mM 5-FU for 72 and 96 hours showed signs of distress with rupture of mitochondrial cristae, dilatation of the cisternae of the endoplasmic reticulum, and in cardiomyocytes, numerous autophagic vacuoles as compared to controls (growth medium and growth medium with DMSO vehicle). To further analyze induction of autophagy, acidic vesicular organelles (AVOs), which consist of autophagosomes and autolysosomes, were quantified by flow cytometry after cell staining with AO, a weak base that accumulates in acidic areas (Fig. 4). A time- and dose-dependent, statistically significant accumulation of AVOs in cardiomyocytes during 5-FU treatment was found (Fig. 4A). In contrast, endothelial cells generally did not show an increase in AVOs with 5-FU treatment (Fig. 4B).

**Figure 5:**
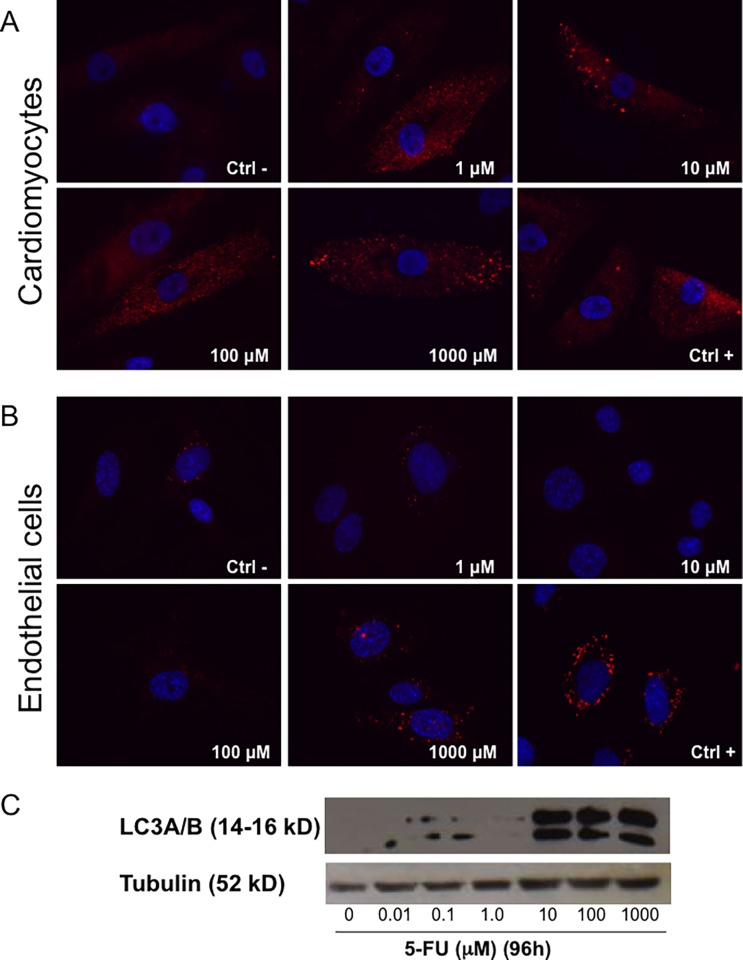
LC-3 expression as autophagic marker induced by 5-FU treatment. Staining with anti-LC3 showed punctae formation associated with the induction of the autophagic process. HCM (A) at all concentrations evaluated showed presence of LC-3 positive vesicles, while in HUVEC (B) the presence of these structures were not detectable unless using the highest 5-FU concentration. Cloroquine used as a positive control (Ctrl+) clearly induced vesicle formation. (C) Western blot analyses confirmed LC3 expression and increase on HCMs at the higher concentration of the drug.

To confirm the establishment of an autophagic process in cells treated with 5-FU, we stained HCMs and HUVE cells with an anti-LC3 antibody (Fig. 5), a major constituent of the autophagosome. This double membrane structure sequesters the target organelle/protein and then fuses with endo/lysosomes where the contents—and LC3—are degraded. Confirming the results obtained with AO, LC3 staining was detected in 5-FU-treated HCMs (Fig. 5A) at all concentrations analyzed (1 μM to 1000 μM). In endothelial cells (Fig. 5B) only a light, diffused presence of the cytoplasmic form of LC3 was detected, LC3-associated punctae were only observed at the highest dose (1000 μM). The positive control (10 μM Cloroquine) clearly showed accumulation of LC3 punctae both in HUVECs and HCMs. These data were also confirmed using western blot analysis for LC3 expression in HCMs (Fig. 5C).

### 5-FU induces reactive oxygen species generation

**Figure 6:**
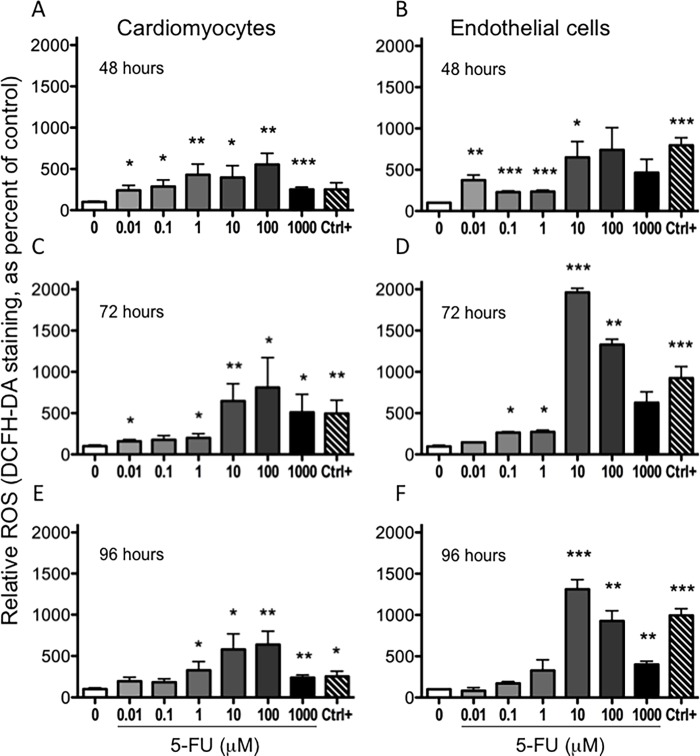
Induction of reactive oxygen species by 5-FU. DCFH-DA stained cells revealed an increased ROS production after 48, 72 and 96 hours of treatment with increasing concentrations of 5-FU. The percentage of cells positive for ROS production was statistically significant at several concentrations examined for both cell types (A, B). *P<0.05; **P<0.01; ***P<0.005. 4-HPR and 0.2% DMSO (vehicle) were used as positive and negative controls. Mean ± S.E.M. of four different experiments for each cell type is shown.

**Figure 7:**
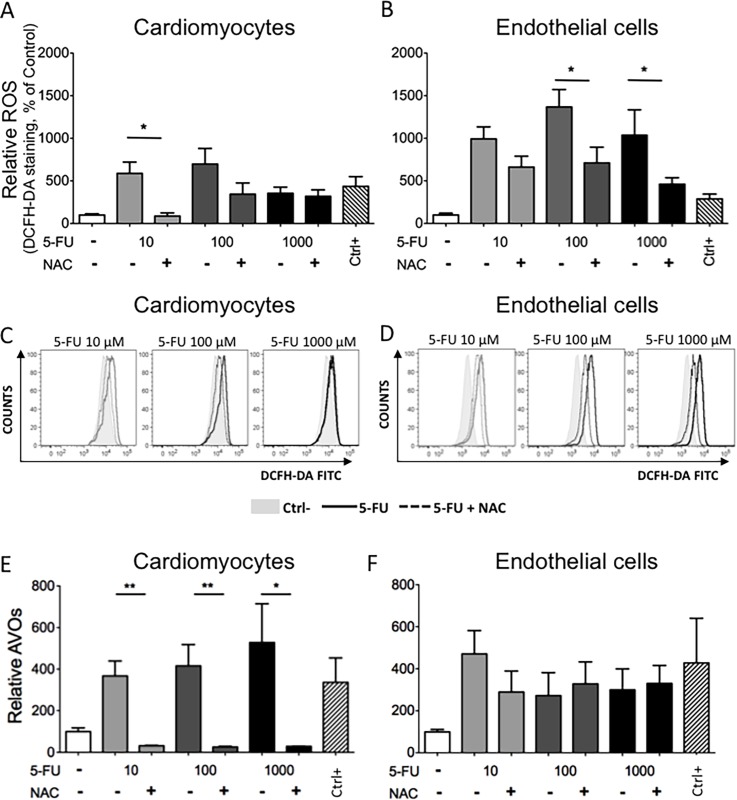
Effects of a ROS scavenger on ROS and AVOs formation. HCM and HUVE cells treated with 5-FU at the indicated concentrations together with the ROS scavenger NAC (10 mM) resulted in a reduction of ROS production. Both relative ROS as percentage of negative control (A, B) and representative histograms of flow cytometric data (C, D) are shown; light grey histograms 10 μM 5-FU, dark grey histograms 100 μM 5-FU, black histograms 1000 μM 5-FU; grey shaded histograms = negative control (Ctrl-); solid lines = 5-FU; dotted lines = 5-FU+NAC. NAC also reduced formation of AVOs in HCM (E), suggesting that ROS is also involved in the autophagic response in these cells. Since endothelial cells did not show induction of AVOs, NAC has little effect on AVO production in these cells (F). *P<0.05; **P<0.01. Mean ± S.E.M. of three different experiments for each cell type is shown.

Reactive oxygen species (ROS) are often released by cells in response to chemotherapeutic drugs and are cytotoxic [42]. We investigated intracellular ROS production in HCMs and endothelial cells using DCFH-DA staining, a cell penetrating dye that becomes fluorescent after reacting with ROS. We observed a dose- and time-dependent generation of ROS in both cell types (Fig. 6). In basal conditions both cell types showed a comparable low level of ROS. When treated with 5-FU, HUVE and HCM cells showed a concentration dependent, statistically significant elevated ROS production, reaching peaks 10 to 100 μM 5-FU at all time points assessed. There was a statistically significant increase in ROS production (Fig. 6) at 5-FU doses below the EC_50_ (Fig. 1) for both HCM and HUVE cells. ROS production was confirmed using the ROS scavenger NAC [43]. Contemporary treatment with 5-FU and NAC significantly reduced ROS levels for both HCM and HUVEC (Fig. 7A-D). We then examined the effect of NAC on AVOs formation as a surrogate for autophagy. NAC significantly inhibited AVOs formation in cardiomyocytes (Fig. 7E). Since the HUVEC response to 5-FU did not involve AVOs (Fig. 4B), NAC was also ineffective at modulating AVOs levels (Fig. 7F). Influence of NAC and 5-FU co-treatment was also evaluated on the HCT116 CRC cell line, where NAC enhanced the cytotoxicity of 5-FU toward the tumor cells (data not shown).

### Induction of senescence by 5-FU

**Figure 8:**
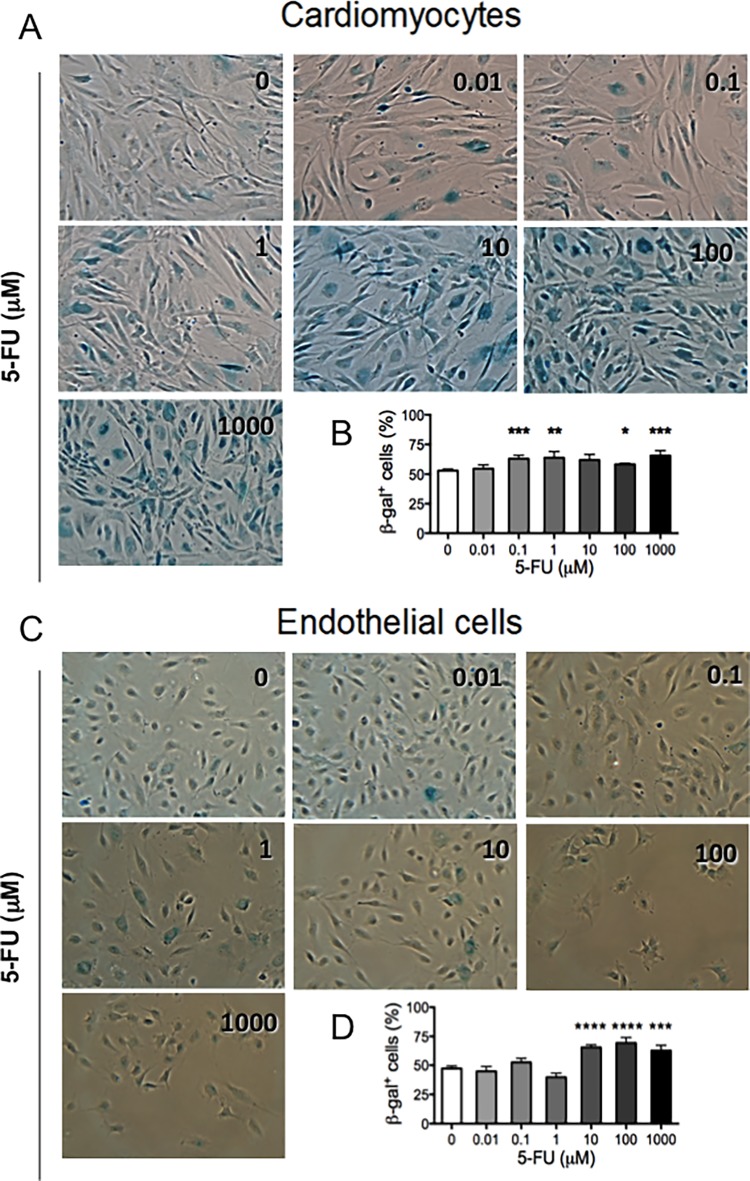
Senescence induction by 5-FU. After 72 hours of treatment with 5-FU (shown in μM), both cell types display a dose-dependent increase in the percentage of senescent cells. Representative images of β-GAL-stained blue-senescent cells are shown for 5-FU or DMSO treated HCMs (A) and endothelial cells (C). The ratio between the β-galactosidase positive blue-stained cells and total nuclei was calculated for each observed field and the results reported as mean of percentages of senescent cells per treatment (B, D). Five fields per conditions were observed; mean ± S.E.M. of three different experiments for each cell type is shown. (*P<0.05; **P<0.01; ***P<0.005; ****P<0.0001).

ROS production is associated with senescence induction, in particular in endothelial cells [44,45]. Replicative senescence or stress-induced premature senescence has been described *in vitro* [46]. This latter type of senescence process has been associated with drugs acting on DNA, including 5-FU [47–49]. Senescent endothelial cells are characterized by increased activity of senescence-associated β-galactosidase (β-GAL) and the senescence-associated secretory phenotype associated with atherosclerosis and vascular dysfunction [46,50]. Previous studies have suggested that physiologically senescent cells also loose their angiogenic potential [51]. Given the generation of ROS by 5-FU, we investigated senescence induction using β-GAL activity, which is readily detectable in senescent cells, yet undetectable in quiescent, immortal or tumor cells. After 72 hours of treatment with 5-FU, the numbers of total and senescent cells per field were counted and results reported as percentage of senescent cells per concentration. Both cell types showed a dose dependent statistically significant increase in the percentage of senescent cells compared to control (Fig. 8). HUVE cells showed significantly increased average percentages of senescent cells at the higher drug concentrations, from 47% in untreated control to 66%, 69% and 63% in 10, 100 and1000 μM, respectively. This was accompanied by an absolute reduction in total HUVE cell number with a relative increase in senescent cells (visible in bright field pictures, Fig. 8C). In HCM, the basal level of senescent cells was higher than endothelial cells (53%, physiologically expected) and the greater sensitivity to β-GAL staining is evident starting at low concentrations of 5-FU. The number of β-GAL stained cells (Fig. 8B) was significantly increased for all the concentrations from 0.1 to 1000 μM except for the 10 μM point (63%, P<0.005; 64%, P<0.01; 62%, not significant; 58%, P<0.05; 66%, P<0.005 respectively, as compared to control). Our data showed that induction of senescence could be considered an additional mechanism by which 5-FU impairs cells function, leading to vascular collapse or vasospasm associated with 5-FU cardiac toxicity.

### Cardiovascular toxic effects of 5-FU *in vivo*

**Figure 9:**
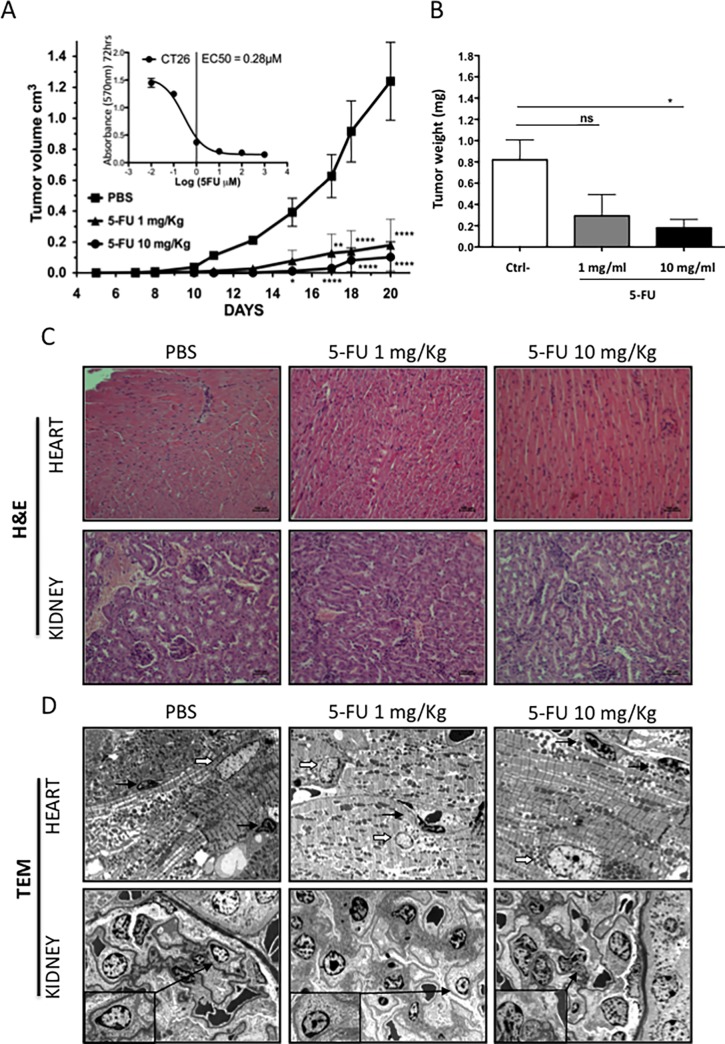
*In vivo* effect of 5-FU. The CT26 murine colon adenocarcinoma cell line was used to evaluate effect of 5-FU *in vivo* on BALB/c mice. Sensitivity to 5-FU was demonstrated both *in vitro* in MTT (A, inset) and *in vivo* (A, B). Both treatment schedules (1 mg/kg every day, 10 mg/kg every other day) significantly reduced tumor growth (A) and weight upon sacrifice (B) as compared to control (PBS). Hematoxylin/eosin staining of renal and cardiac tissues from PBS and 5-FU treated animals (C). Transmission electron microscopy of renal tissues showed alterations (cytoplasmic vacuolization, membrane breakage) of endothelial cells (insets) in both treated groups with respect to controls (D). Cardiac tissues showed intact cardiomyocytes (nuclei indicated by thick white arrows) and occasional minor alterations of endothelial nuclei (thin black arrows) at the 10 mg/kg dose.

We initially evaluated the sensitivity murine colon adenocarcinoma cell line CT26 to 5-FU *in vitro* using the MTT assay (Fig. 9A inset). *In vivo*, treatment with 5-FU given every day (1 mg/kg) or every two days (10 mg/kg) showed a significant inhibition of tumor growth (Fig. 9A) and of tumor weight (Fig. 9B). Hematoxylin/eosin staining did not show any evident alterations of the cardiac or renal tissues (Fig. 9C). In heart tissues only minor alterations of endothelial nuclei were detected in the 10 mg/kg treatment group which might be a sign of endothelial cell stress. The cardiomyocytes appeared unaffected. Transmission electron microscopy indicated cytoplasmic vacuolization and membrane breakage of endothelial cells in renal tissues (Fig. 9D), an organ often involved in early symptoms of cardiovascular toxicity due to anticancer drugs [52,53].

## Discussion

There is increasing awareness that toxicity to the cardiovascular (CV) system limits treatment efficacy and affects the quality of life of the cancer survivor [16–18,32,35]. Anthracyclines are known to cause CV toxicity, in particular in combination with trastuzumab [16–18,32], which by itself has some degree of cardiotoxicity [16–18,32,54]. Reports of cardiac toxicities of fluoropyrimidines (5-FU and its prodrugs) are increasing although a conclusive explanation of these effects is still lacking. To shed additional light on this, we investigated the effects of 5-FU on human endothelial cells and cardiomyocytes, key components of the CV system both of which are affected by fluoropyrimidine chemotherapy. There are several limitations to this study, including the use of human *in vitro* models and relatively “young” murine *in vivo* models. However, we feel these data do reflect some potential clinical scenarios in patients treated with 5-FU and its pro-drugs.

The concentrations of 5-FU used here mimic those of several clinical studies [24,25], bolus intravenous administration results in plasma peaks up to the millimolar range with a rapid decline, while with continuous infusions of 5-FU were in the 1.4–6.7 μM range. Infusion with Capecitabine resulted in therapeutic dosing between 3.46 and 6.53 μM [55]. Further, the EC_50_ of effects on cardiomyocytes and endothelial cells was similar to that of CRC cell lines *in vitro*. 5-FU affected that proliferative capacity of both cardiac myocytes and endothelial cells, in agreement with previous studies [56–59].

A decrease in the cycling rate might be a mechanism of tumor 5-FU escape [60], thus it could be hypothesized that slow replicating potential could be a protection against the effects of 5-FU on normal cells. However, our data suggest that this is not the case, where we observed a decrease in endothelial cells in S and G2/M, while in cardiomyocytes we found a slight increase in G2/M phase, and a complete absence of replicating cells at higher 5-FU concentrations. The dissimilar behavior (e.g. accumulation in diverse phases of cell cycle) of different cell types in response to 5-FU is not surprising, tumor cells of various histotypes showed different cell cycle distribution after 5-FU treatment [61].

Although having a limited replicative potential, cardiomyocytes were quite sensitive to 5-FU. Consistent with recent publications investigating primary and immortalized rat cardiomyocyte cell lines [55–57], we detected induction of free radicals and oxidative stress after 5-FU treatment of primary human cardiac cells. We also observed that cardiomyocytes were more predisposed to enter autophagy as compared to endothelial cells. We previously reported induction of mitochondrial autophagy in rat cardiomyocytes treated with 5-FU [62]. Several publications have shown that autophagy is an escape mechanism that colon cancer cells perform during 5-FU treatment [63–65], and the interruption of the autophagic process enhances tumor cell sensitivity to the drug. Autophagy is a conserved process that keeps a constant balance between biosynthetic and catabolic processes [66–68]. Although autophagy promotes a cell-survival response, morphological features of autophagy have also been observed in dying cells, although whether it is the cause of death or simply a stress response is controversial [69]. Autophagy, apoptosis and senescence can be induced by ROS elevation [70–72], and the ROS scavenger NAC was able to revert induction of autophagic markers in cardiomyocytes. Taken together with previous demonstrations of 5-FU smooth muscle cell alterations [39], these results indicate that 5-FU has substantial effects on the cardiovascular system. Further, the indication that the TP enzyme is up-regulated in atherosclerotic plaques [30] and during myocardial damage [31], suggest that 5-FU and its prodrugs could directly damage the myocardium, in particular in older cancer patients. *In vivo* studies using doses of 5-FU that inhibited tumor growth are clearly associated with damage to renal endothelial cells, with TEM analysis showing cytoplasmic vacuolization and membrane breakage, results in line with prevous reports [73].

The absence of damage in heart tissues found *in vivo* could be explained by the relatively young age of mice, which may not represent the aged cardiomyocytes in most oncologic patients. However, renal toxicity is a growing concern closely related to cardiovascular toxicity [52,53].

The cardiotoxicity of fluoropyrimidines has been associated with induction of vasospasms and subsequent angina/ischemia [74–77]. Direct endothelial damage could provoke thrombosis and release of vasoactive substances [24] as has been observed *in vivo* [78] although there are contrasting reports regarding the pro-thrombotic activity of 5-FU [79,80]. Cell cycle block, apoptosis and alterations of contractility of smooth muscle cells treated with 5-FU *in vitro* have recently been reported [39]. These data could explain the gastrointestinal side effects of 5-FU, but they may also contribute to the enhanced vasoresponse associated with 5-FU induced angina [12,21,81], in particular in the context of endothelial distress and senescence that we report here. However, a lack of response of angina occurring during 5-FU treatment after vasodilator calcium antagonist administration has been described [82], suggesting other mechanisms as well. In addition, heart failure and myocardiotoxicity have also been reported as complications of 5-FU therapy [12,19–21,82–84]. Another proposed mechanism leading to ischemia is the 5-FU-induced decrease in the oxygen binding capacity of erythrocytes [85], suggesting decreased oxygen transfer capacity or inhibition of eNOS activity by Capecitabine [86]. Previous *in vivo* studies showed the reduction of antioxidant defense capacities [13].

Finally, we observed induction of senescence in both cell types upon exposure to 5-FU. Senescence cells activate signaling pathways that lead to the production and release of cytokines, chemokines and growth factors. Interestingly, senescent cells have been found to induce neighboring cells to enter senescence [87], likely through gap junction communication. Considering the advanced age of the majority of oncologic patients, latent age-compromised heart tissue performance could be further decreased by 5-FU administration.

Our data add new insights into the possible mechanisms involved in at least some of the manifestations of 5-FU cardiotoxicity, and suggest potential prevention strategies to reduce these severe side effects of an otherwise very useful drug family for treatment of numerous cancers.

## Supporting Information

S1 Fig Metabolism of Capecitabine and 5-FU. Capecitabine, an orally administered fluoropyrimidine carbamate 5-FU prodrug, is converted into 5-FU through three sequential steps: it is converted to 5'-deoxy-5-fluorocytidine (5'-DFCR) by carboxylesterase (CES) located in the liver, followed by the conversion of 5'-DFCR to 5'-deoxy-5-fluorouridine (5'-DFUR) by cytidine deaminase (CDA) in the liver and in solid tumors. Finally, in solid tumors 5'-DFUR is converted to 5-FU by thymidine phosphorylase (TP). 5-FU is converted to 5-fluorourodeoxyuridine (5-FUdR) by the action of thymidine phosphorylase (TP). 5-FUdR is then converted by thymidine kinase (TK) to 5-fluorodeoxyuridine monophosphate (5-FdUMP). 5-FdUMP inhibits DNA synthesis by competing with deoxyuridine monophosphate (dUMP) for binding to thymidylate synthase (TS). 5-FU inhibits RNA synthesis, processing and function through a pathway that involves its metabolism by orotate phosphoribosyltransferase (OPRT) to 5-fluorouridine monophosphate (5-FUMP) and subsequent conversion to 5-fluorouridine triphosphate (5-FUTP) via 5-fluorouridine diphosphate (5-FUDP). 5-FU is catabolized and inactivated through sequential enzymatic steps initiated by dihydropyrimidine dehydrogenase (DPD).
(TIFF)

S2 Fig Cytostatic effects of 5-FU on cardiomyocytes and endothelial cells. Raw MTT data are shown indicating the difference in growth of the different cell lines. MTT data at 72 hours were used to calculate the EC_50_ for each cell line, again differences in replication rates are visible. 5-FU concentrations are reported in μM on a Log_(10)_ scale.
(TIFF)

S3 Fig Effects of 5-FU on the cell cycle. PI-staining cumulative histograms of three independent experiments are shown for 96 hours 5-FU treated (10 nM to 1 mM) cells (A). Differences among groups were not statistically significant although evident. The base analogue BrdU was added to HCMs and HUVECs after 84 hours of drug treatment (100 nM to 1 mM) (B). For each dot plot, the bottom gate comprises total BrdU^-^ cells (not proliferating, G1 and G2/M phases), while in the upper quadrant BrdU^+^ cells are proliferating (S phase). Vincristine was used as positive control to arrest proliferation in G2/M-phase.
(TIFF)

S4 Fig Apoptosis detection after 5-FU treatment. Representative histograms showing increase of Annexin-V^+^ in cardiomyocytes and endothelial cells in response to 5-FU concentrations from 10 nM to 1 mM. Vincristine was used as positive control. The effects of different drug concentrations are represented in gray scale. Dotted line: isotype control. Bold black line: vincristine positive control.
(TIFF)
