# Supplementary figures and images for: Effects of 5-Fluorouracil on Morphology, Cell Cycle, Proliferation, Apoptosis, Autophagy and ROS Production in Endothelial Cells and Cardiomyocytes
Source: PLoS One. 2015 Feb 11;10(2):e0115686. doi: 10.1371/journal.pone.0115686 (PMC4324934; doi:10.1371/journal.pone.0115686)

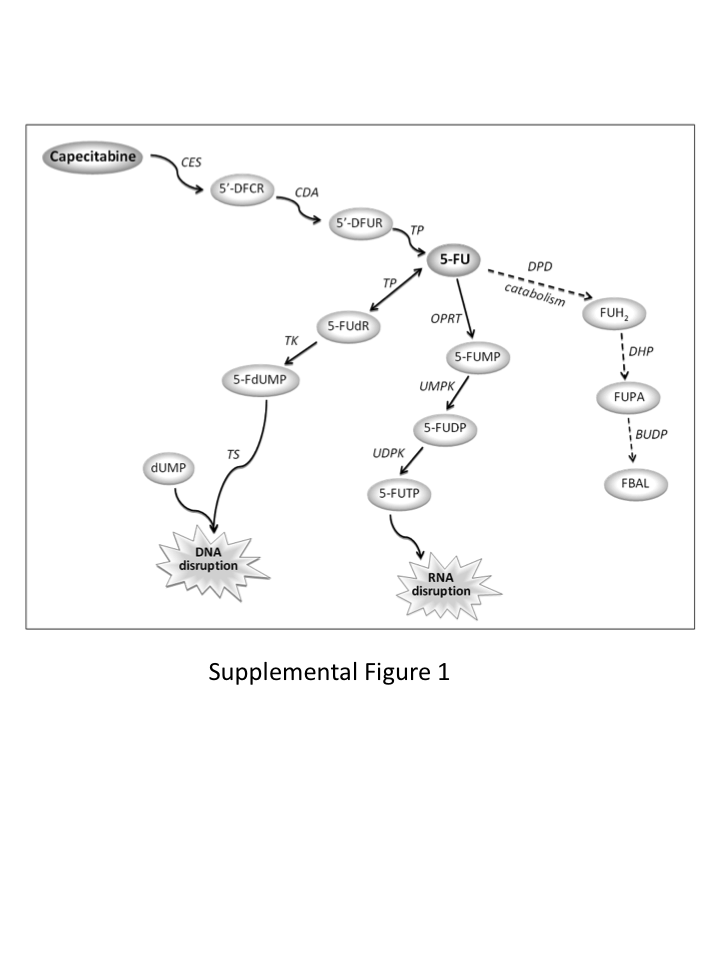

Supplement: S1 Fig — Capecitabine, an orally administered fluoropyrimidine carbamate 5-FU prodrug, is converted into 5-FU through three sequential steps: it is converted to 5'-deoxy-5-fluorocytidine (5'-DFCR) by carboxylesterase (CES) located in the liver, followed by the conversion of 5'-DFCR to 5'-deoxy-5-fluorouridine (5'-DFUR) by cytidine deaminase (CDA) in the liver and in solid tumors. Finally, in solid tumors 5'-DFUR is converted to 5-FU by thymidine phosphorylase (TP). 5-FU is converted to 5-fluorourodeoxyuridine (5-FUdR) by the action of thymidine phosphorylase (TP). 5-FUdR is then converted by thymidine kinase (TK) to 5-fluorodeoxyuridine monophosphate (5-FdUMP). 5-FdUMP inhibits DNA synthesis by competing with deoxyuridine monophosphate (dUMP) for binding to thymidylate synthase (TS). 5-FU inhibits RNA synthesis, processing and function through a pathway that involves its metabolism by orotate phosphoribosyltransferase (OPRT) to 5-fluorouridine monophosphate (5-FUMP) and subsequent conversion to 5-fluorouridine triphosphate (5-FUTP) via 5-fluorouridine diphosphate (5-FUDP). 5-FU is catabolized and inactivated through sequential enzymatic steps initiated by dihydropyrimidine dehydrogenase (DPD). (TIFF) [file pone.0115686.s001.tiff]

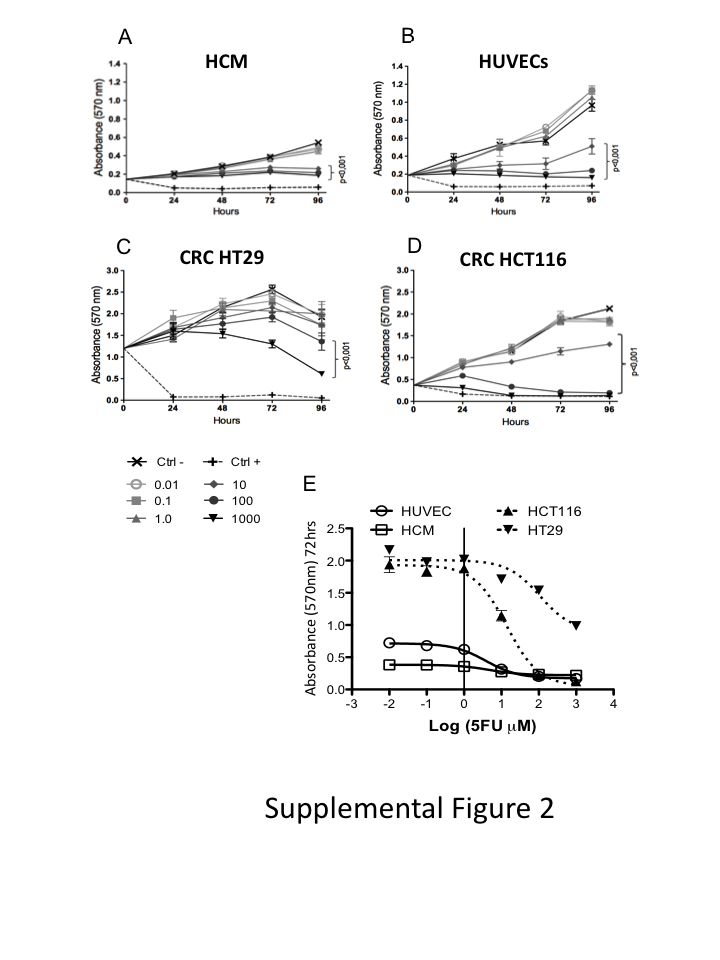

Supplement: S2 Fig — Raw MTT data are shown indicating the difference in growth of the different cell lines. MTT data at 72 hours were used to calculate the EC50 for each cell line, again differences in replication rates are visible. 5-FU concentrations are reported in μM on a Log(10) scale. (TIFF) [file pone.0115686.s002.tiff]

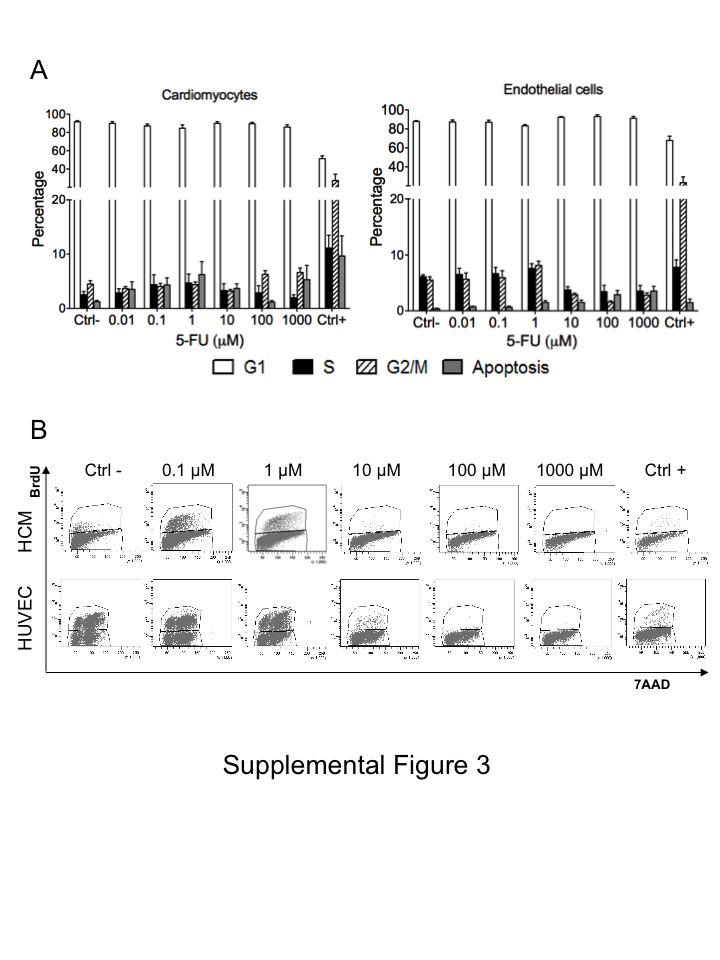

Supplement: S3 Fig — PI-staining cumulative histograms of three independent experiments are shown for 96 hours 5-FU treated (10 nM to 1 mM) cells (A). Differences among groups were not statistically significant although evident. The base analogue BrdU was added to HCMs and HUVECs after 84 hours of drug treatment (100 nM to 1 mM) (B). For each dot plot, the bottom gate comprises total BrdU- cells (not proliferating, G1 and G2/M phases), while in the upper quadrant BrdU+ cells are proliferating (S phase). Vincristine was used as positive control to arrest proliferation in G2/M-phase. (TIFF) [file pone.0115686.s003.tiff]

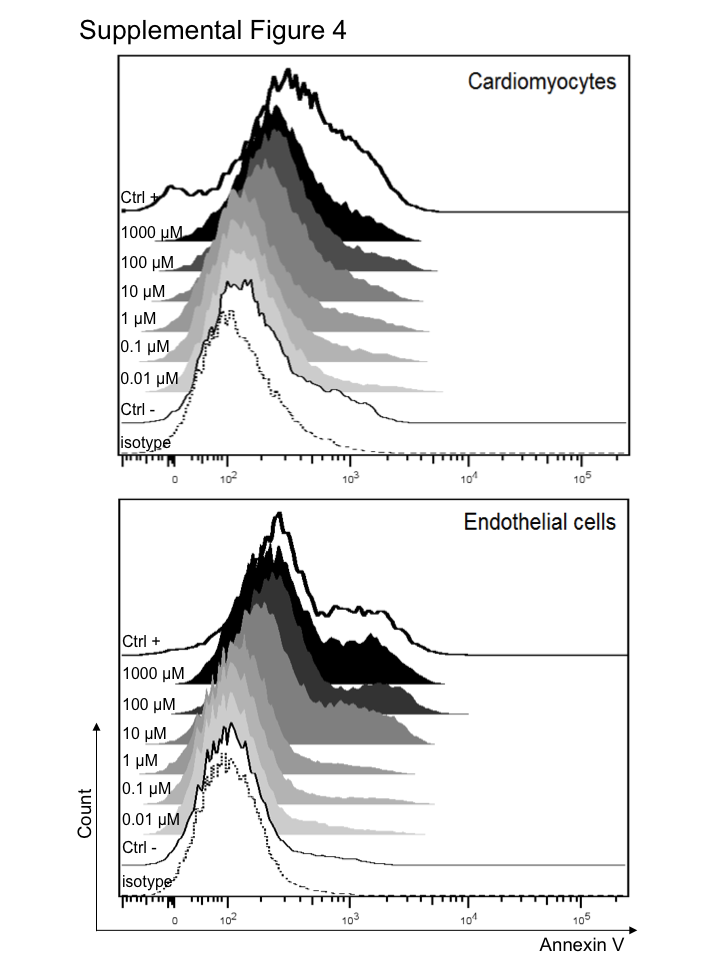

Supplement: S4 Fig — Representative histograms showing increase of Annexin-V+ in cardiomyocytes and endothelial cells in response to 5-FU concentrations from 10 nM to 1 mM. Vincristine was used as positive control. The effects of different drug concentrations are represented in gray scale. Dotted line: isotype control. Bold black line: vincristine positive control. (TIFF) [file pone.0115686.s004.tiff]
